# Multidimensional regulatory architecture of endometrial receptivity and its translational implications for implantation failure

**DOI:** 10.3389/fendo.2026.1892624

**Published:** 2026-07-27

**Authors:** Fengping Zhang, Qunhong Fang, Rui Ma, Lin Ye, Yang Ke, Haishan Zheng, Hao Cui, Xinying Huang, Lingchuan Li, Xi Luo

**Affiliations:** 1 Department of Reproductive Medicine, National Health Commission (NHC) Key Laboratory of Healthy Birth and Birth Defect Prevention in Western China, the First People’s Hospital of Yunnan Province, School of Medicine, Kunming University of Science and Technology, Kunming, China; 2 Department of Reproductive Medicine, National Health Commission (NHC) Key Laboratory of Healthy Birth and Birth Defect Prevention in Western China, the First People’s Hospital of Yunnan Province, Kunming University of Science and Technology Affiliated Hospital, Kunming, China; 3 Department of Gynecology, the First People’s Hospital of Yunnan Province, Kunming University of Science and Technology Affiliated Hospital, Kunming, China

**Keywords:** embryo implantation, endometrial receptivity, epigenetic regulation, hormonal signaling, immune tolerance, multidimensional regulatory network

## Abstract

Endometrial receptivity within the window of implantation (WOI) has been increasingly recognized as an independent determinant of pregnancy outcome in assisted reproduction. Persistent recurrent implantation failure (RIF) is observed in a substantial subset of cycles even after transfer of high-quality embryos, which points toward endometrial factors as targets for closer investigation. Mechanistic work has predominantly been confined to individual signaling pathways examined in isolation, and convergence of hormonal, immune, and epigenetic systems on the receptive state remains insufficiently characterized. Structural readouts dominate at the clinical interface, where endometrial thickness and Doppler indices afford limited insight into molecular dynamics. Consistent clinical benefit has not been delivered by transcriptomic-guided transfer strategies across randomized controlled trials. Evidence is synthesized here across three regulatory tiers of receptivity control. The hormonal signaling network centered on the estrogen and progesterone axis is reviewed together with auxiliary endocrine modulators. The immune regulatory network is dissected through uterine natural killer (uNK) cells, regulatory T (Treg) cells, and macrophage phenotypic transitions. The epigenetic layer is addressed at the level of DNA methylation, histone modification, and non-coding RNA activity. Cross-axis coupling is then examined, with leukemia inhibitory factor (LIF) positioned as a convergence node that integrates endocrine input with immune output and chromatin state. Imaging, molecular profiling, and liquid biopsy are each appraised against current evidence. Translational obstacles imposed by the phenotypic heterogeneity of implantation failure are then considered. Receptivity emerges as a product of coordinated multi-tier regulation rather than as the output of any single pathway. Mechanism-informed stratification, integrating mechanistic evidence with multimodal patient data, lies at the next frontier of clinical translation.

## 1 Introduction

Successful embryo implantation is contingent upon attainment of a transient receptive state by the endometrium during the window of implantation (WOI). This interval typically spans post-ovulatory days 6 to 10, corresponding approximately to days 20 through 24 of an idealized 28-day menstrual cycle, with documented inter-individual variation in WOI timing (1, 2). With expansion of assisted reproductive technologies (ART), endometrial receptivity has been increasingly recognized as a contributor to recurrent implantation failure (RIF) independent of embryo quality (3, 4).

Mechanistic investigation of receptivity has been predominantly anchored in estrogen and progesterone signaling through their cognate receptor pathways. Such single-pathway frameworks remain informative yet insufficient to capture the multidimensional coordination of immune and epigenetic processes underlying receptivity establishment (5). Intercellular communication forms an additional regulatory layer. Contributions from immune cell populations to localized immune tolerance within the endometrium have been increasingly characterized. Uterine natural killer (uNK) cells and regulatory T (Treg) cells are identified as key mediators. Recruitment and functional activation of these cells are governed by hormonal cues and chemokine gradients, with cytokine networks providing additional input. Epigenetic mechanisms encompassing DNA methylation and histone modifications have been shown to modulate expression of receptivity-associated genes such as the progesterone receptor gene (PGR) and HOXA10 (6, 7). Through these modifications, receptivity is shaped at the transcriptional level.

Clinical assessment methodology has continued to evolve, yet conceptual alignment between evaluation strategies and underlying molecular mechanisms remains incomplete. Conventional parameters, such as endometrial thickness and pulsatility index (PI), primarily capture structural alterations (8, 9). Histological scoring is similarly constrained in its capacity to reflect the dynamic molecular architecture of post-ovulatory endometrial transformation. To address this gap, exosomal miRNA profiling and uterine fluid omics have been explored as non-invasive tools for direct interrogation of functional receptivity markers (10–12). Ultrasound elastography has also been evaluated as an adjunctive modality that assesses receptivity indirectly through quantification of tissue mechanical properties (13).

This review synthesizes three core regulatory dimensions of endometrial receptivity, spanning hormonal signaling, immune modulation, and epigenetic control. Published evidence indicates that these axes jointly construct the functional architecture of the WOI, and that pathological states arise when dysregulation spans several of them at once. Translation is emphasized throughout, with attention directed toward emerging non-invasive molecular biomarkers. Immune mediators and epigenetic regulatory targets are also considered as candidate biomarkers. A conceptual framework for mechanism-informed reproductive management strategies is provided.

## 2 Integrated hormonal signaling regulation as the central axis network driving endometrial receptivity

Endometrial receptivity emerges through a temporal sequence of hormonal events rather than simultaneous multi-signal input. Estrogen initiates the proliferative program, in which ERα activates Wnt/β-catenin and PI3K/AKT cascades that drive epithelial and stromal expansion (14–16). Progesterone then drives the transition from proliferation to differentiation. Signaling through the progesterone receptor (PR) isoforms PR-A and PR-B, together with the cAMP/PKA-GREB1-FOXO1 axis, induces decidualization (17–19). The ratio of PR-A to PR-B determines transcriptomic specificity of the progesterone response, and this ratio is itself subject to DNA methylation control (20, 21). Premature progesterone elevation or epigenetic dysregulation of PGR-encoded PR isoform expression can advance WOI closure, indicating that the timing mechanism is vulnerable to perturbation at both the endocrine and chromatin levels (22). The estrogen-to-progesterone switch therefore times receptivity through coordinated action between the steroid microenvironment and its genomic substrates (7, 23–26).

Once the progesterone-driven differentiation program is underway, embryo-derived and pituitary signals amplify and refine the endometrial response. Human chorionic gonadotropin (hCG) activates LHCGR/PI3K/AKT/eNOS and ERK1/2 pathways, upregulating HOXA10 and vascular endothelial growth factor (VEGF) while inducing matrix metalloproteinase 9 (MMP9) to enhance local blood perfusion (27–29). A separate action of hCG is downregulation of DNA methyltransferase 1 (DNMT1), which reduces methylation at the HOXA10 promoter and thereby locks in HOXA10 accessibility beyond the duration of the hCG stimulus itself (30). Growth hormone (GH) contributes through upregulation of leukemia inhibitory factor (LIF) and engagement of the JAK1/STAT3-SOCS1 feedback loop, a pathway shared with immune signaling (31, 32). Rather than merely reinforcing the progesterone message, hCG and GH extend it into adjacent regulatory domains. hCG deposits an epigenetic mark and GH activates an immune-relevant cytokine, so amplification of the hormonal signal already reaches beyond the classical endocrine circuit into the epigenetic and immune layers.

Beyond the reproductive endocrine axis, systemic metabolic and stress-related hormones modulate the sensitivity of the decidualizing endometrium. The thyroid hormone triiodothyronine (T3), whose intracellular availability is governed by type 2 deiodinase (DIO2), contributes to endometrial receptivity through T3-thyroid hormone receptor (THR) signaling that regulates epithelial cell membrane fluidity and functional responsiveness (33). Melatonin, acting through SIRT1/PI3K/AKT, regulates HOXA10 in bovine endometrial epithelial cells (34) and has been linked to uterine immune microenvironment maintenance during early pregnancy in mice (35). Epinephrine exerts concentration-dependent biphasic effects. Physiological levels promote FOXO1 nuclear localization and decidualization markers through ADRA2C/AKT, whereas pathological elevation suppresses receptivity through receptor downregulation and α-adrenergic vasoconstriction (36, 37). Androgens, acting via androgen receptor (AR) expressed in both epithelial and stromal compartments, upregulate PR expression and engage PI3K/AKT signaling, consistent with a sensitizing role for progesterone action (38–40). What unites these apparently disparate factors is that each adjusts the gain of the progesterone response rather than initiating an independent signaling program, so systemic metabolic and neuroendocrine input reaches the decidualizing endometrium through this shared route.

**Figure 1:**
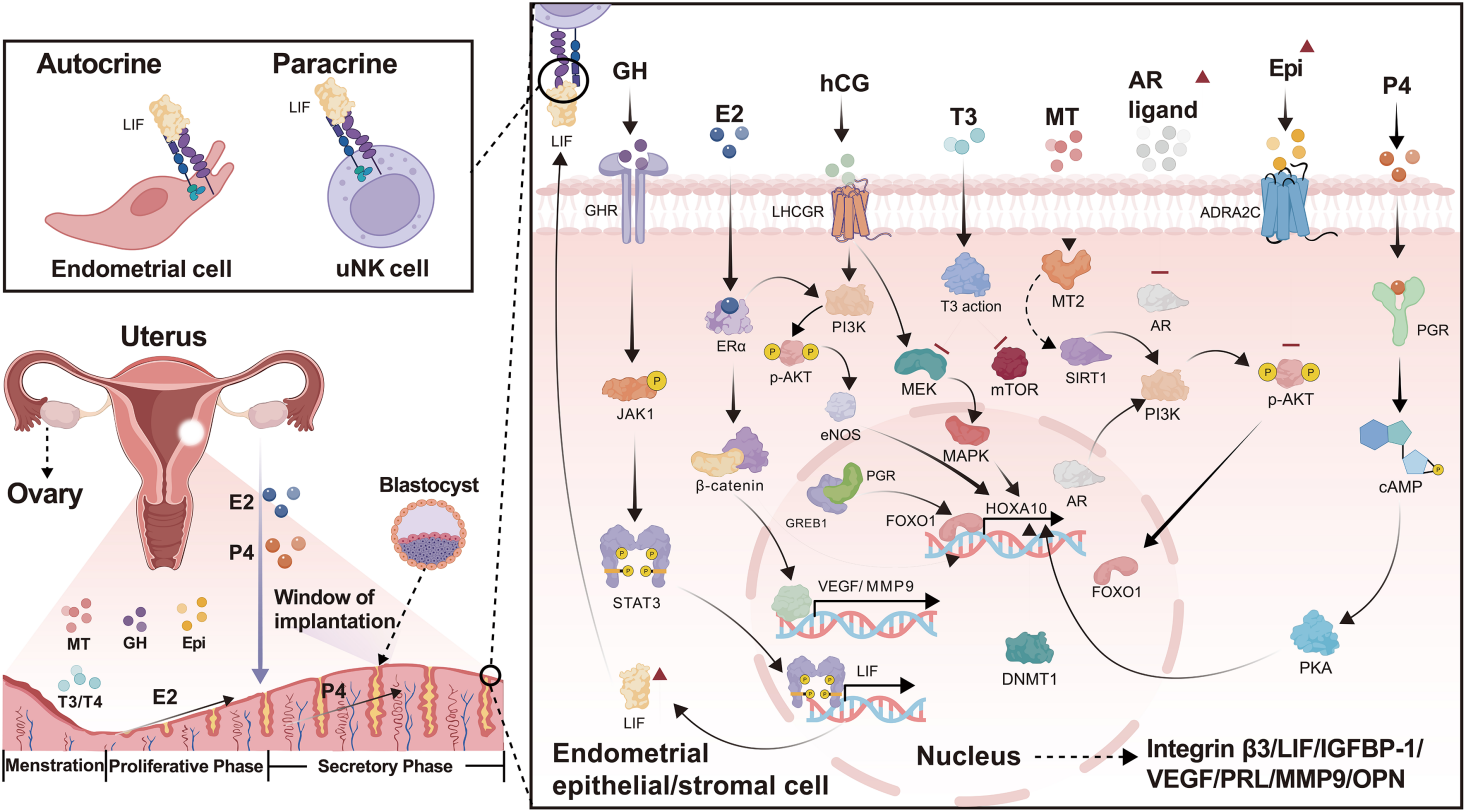
Integrated hormonal signaling architecture of endometrial receptivity. Temporal coordination of the ovarian-uterine endocrine axis across the menstrual cycle is shown on the left, with the implantation window defined by sequential E2 and P4 dynamics. Intracellular signaling cascades activated by multiple hormonal inputs and their convergence on receptivity-associated transcriptional targets are shown on the right.

Hormonal regulation of receptivity thus operates as a temporally organized, multi-tiered network (**Figure 1**). The estrogen-progesterone switch provides the time gate; embryo-derived and pituitary factors amplify and extend the response into epigenetic and immune territory; systemic metabolic hormones tune the sensitivity of the entire apparatus, together laying the structural and functional foundation for implantation.

## 3 Cooperative construction of the receptivity-associated immune environment by immune cells and signaling factors

This hormonal foundation cannot, on its own, secure implantation. Acceptance of the embryo as a semi-allogeneic graft requires a specialized immune-tolerant microenvironment that hormonal signals alone cannot construct. The immune contribution to endometrial receptivity is not a matter of generalized suppression. What the implanting embryo requires is a selective tolerogenic microenvironment that simultaneously permits controlled tissue remodeling, vascular adaptation, and trophoblast invasion while preventing allograft rejection. Building this environment is a phased process in which distinct immune cell populations act in temporal sequence, each executing a defined function before handing off to the next (41). The following paragraphs trace this sequence from vascular preparation through tolerance establishment to dynamic maintenance.

The first phase of this sequence is vascular remodeling driven by uNK cells (42). The predominant uNK phenotype during the WOI is CD56^bright^ CD16^dim/-^, characterized by limited cytotoxicity and strong cytokine secretory capacity (42). These cells drive spiral artery remodeling through paracrine secretion of VEGF-C and Angiopoietin-1/2 rather than through direct cytotoxic mechanisms (43). Single-cell analyses have revealed multiple functional uNK subpopulations, and the immune modules that promote angiogenesis and trophoblast invasion are already pre-configured during the WOI (43, 44). Tissue residency and functional execution depend on autophagy mechanisms in endometrial stromal cells (45), while KIR-HLA-C/HLA-G receptor-ligand interactions fine-tune uNK activation thresholds and secretory profiles to maintain vascular remodeling and trophoblast invasion within controlled boundaries (46).

With the vascular foundation in place, immune tolerance programming becomes the dominant task. Treg cells construct an embryo-protective barrier through secretion of IL-10 and TGF-β (5). CXCL12-CXCR4 signaling recruits Treg cells to the endometrium while concurrently regulating integrin β3 and osteopontin expression to enhance embryo-endometrium adhesion (47). Elevated Th17/Treg ratios in secretory-phase endometrium of RIF patients, accompanied by increased TNF-α and IL-6, confirm that disruption of this balance impairs receptivity (48, 49). Effector T cells are not entirely silenced during this tolerance program. Transient Th1/Th17 signaling contributes to pre-implantation tissue remodeling and immune pre-activation (5, 50), and implantation failure correlates more closely with delayed termination of the inflammatory phase than with the presence of pro-inflammatory cells themselves (51).

Macrophages provide the dynamic maintenance layer that sustains WOI stability across the implantation window. During early WOI, macrophages with an M1-like profile produce IL-1β and TNF-α alongside matrix metalloproteinases that remodel extracellular matrix and facilitate trophoblast invasion (52, 53). After implantation, a phenotypic shift toward M2-enriched profiles yields IL-10 and TGF-β, which sustain maternal-fetal immune tolerance and decidual tissue repair (54). CX3CL1-CX3CR1 signaling and granulocyte colony-stimulating factor (G-CSF)-activated PI3K/AKT pathways coordinate this transition (55–57). Single-cell data indicate that endometrial macrophages conform to a continuous functional spectrum rather than a strict M1/M2 dichotomy, with co-expression of pro-inflammatory and repair-associated genes observed in certain WOI macrophages (53, 58). Temporal dysregulation of state switching, rather than polarization abnormality per se, may be the critical determinant of implantation window stability. The IL-33/ST2 axis, a communication link between stromal cells and the immune system, provides temporally regulated signaling essential for WOI maintenance and prevention of endometrial fibrosis (59–61).

The same uNK cells that enable vascular remodeling can become detrimental when subpopulation balance shifts. In certain RIF patients, pro-inflammatory signaling involving IFN-γ is abnormally activated in uNK subpopulations, accompanied by KIR-HLA receptor-ligand matching imbalances (43, 44, 46). This shift from regulatory toward inflammatory phenotypes disrupts immune interactions between endometrium and embryo. The implication is that cell identity alone does not determine functional outcome; the same cellular population can promote or obstruct implantation depending on the cytokine context and receptor-ligand configuration it encounters. Function, in the endometrial immune system, is context-dependent rather than lineage-fixed.

**Figure 2:**
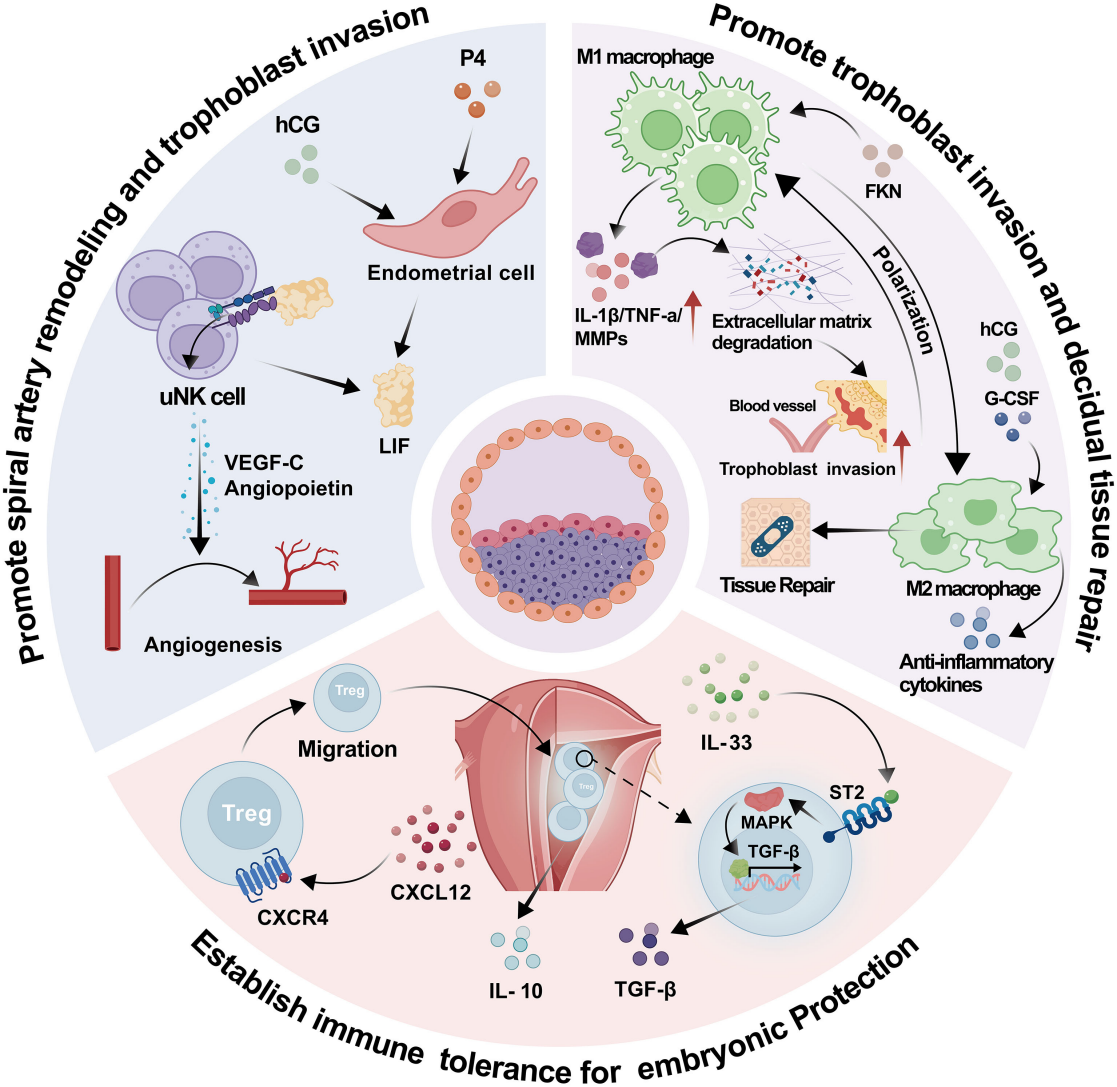
Immune cell coordination of the receptive endometrial microenvironment. Cooperative contributions of uNK cells, macrophages, and Treg cells to the peri-implantation immune microenvironment are illustrated. Spiral artery remodeling and trophoblast invasion are mediated by uNK cells through angiogenic and cytokine outputs. Macrophages undergo M1-to-M2 phenotypic transitions by which extracellular matrix remodeling is balanced with tissue repair and immune tolerance. Treg cells are recruited via CXCL12-CXCR4 chemotaxis and maintain maternal-fetal tolerance through anti-inflammatory mediators. IL-33/ST2 signaling bridges stromal and immune compartments.

Across the temporal sequence described above, LIF and VEGF emerge as signal convergence points where hormonal and immune information intersects. LIF secretion by uNK cells is regulated by both embryo-derived hCG and progesterone-dependent pathways (32, 62, 63); it activates STAT3 signaling that synergistically promotes implantation while feeding back onto immune cell polarization. VEGF, similarly induced by hormonal inputs, directs both vascular remodeling and immune cell trafficking (64) (**Figure 2**). These molecules do not belong exclusively to either the hormonal or immune category; they operate at the interface, converting endocrine instructions into localized microenvironment changes. That identical hormonal inputs can produce different cytokine responses in different cells points to a further regulatory layer, one that sets how strongly and how quickly each cell responds. This layer is epigenetic, and it is the subject of the following section.

## 4 Epigenetic regulation as the hidden regulatory layer coordinating hormonal and immune signals

**Figure 3:**
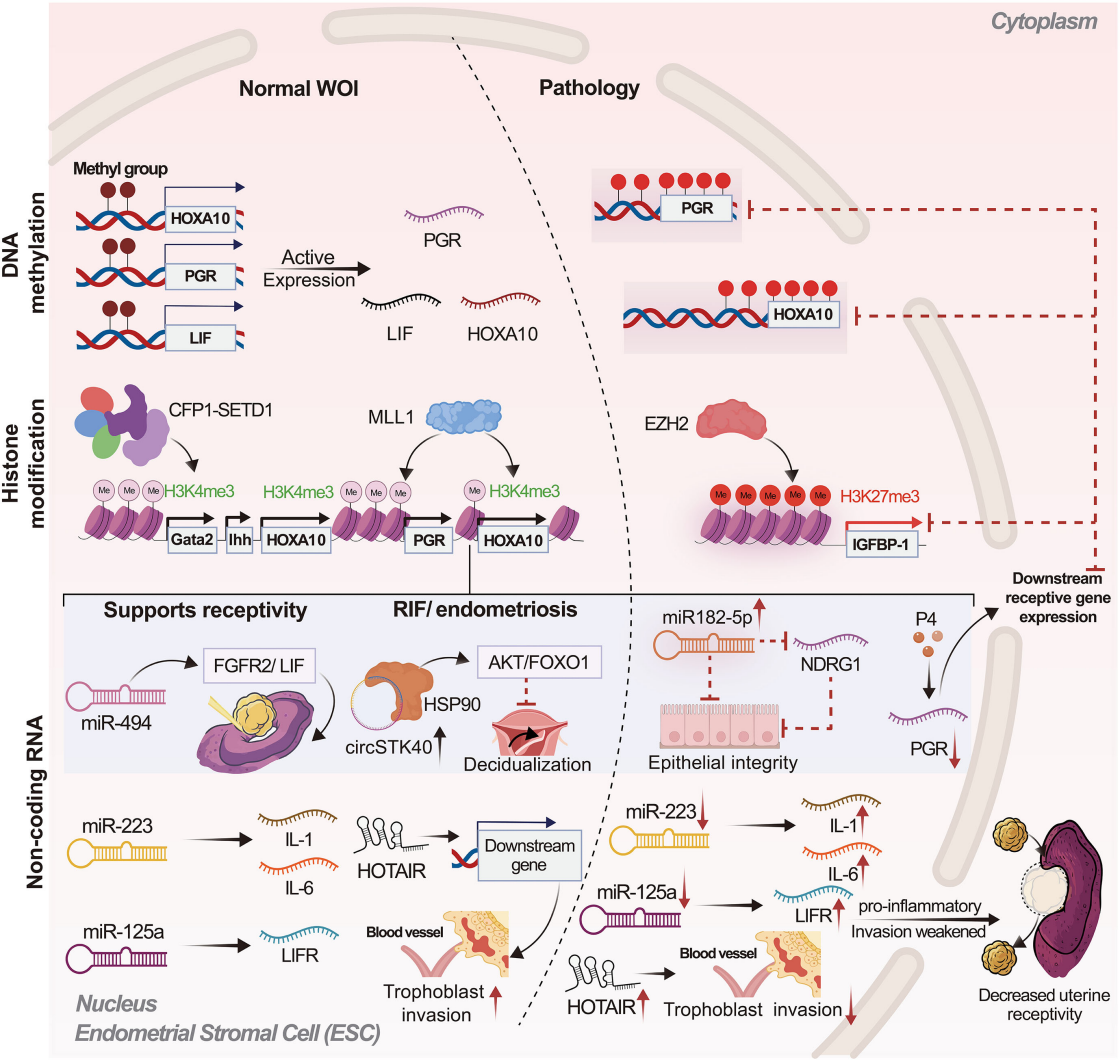
Epigenetic regulatory landscape underlying endometrial receptivity. Three layers of epigenetic regulation are depicted under normal and pathological conditions. Transcriptional accessibility of receptivity genes is controlled by DNA methylation. Histone modifications including H3K4me3 and H3K27me3 tune expression intensity at decidualization-related loci. Non-coding RNAs modulate immune signaling, epithelial integrity, and decidualization at the post-transcriptional level. Disruption across these layers is associated with impaired receptivity and implantation failure. Events depicted occur predominantly in endometrial stromal cells unless otherwise indicated.

The preceding sections established that hormonal signals initiate receptivity and immune cells execute microenvironment remodeling (**Figure 3**). Whether a given cell can respond to these signals at all depends on the accessibility of its chromatin. Epigenetic modifications set this accessibility, opening or closing the genes that hormonal and immune signals target (21, 65–67). A cell whose promoters are silenced cannot transcribe progesterone target genes however high the circulating hormone concentration, and a cell whose enhancers lack activating histone marks cannot mount the cytokine response that immune signals demand. Epigenetic regulation, viewed from this angle, does not form a third independent signaling system; it is the layer that determines whether the other two systems reach their targets. Dynamic chromatin remodeling across the menstrual cycle, mapped at single-cell resolution, confirms that the implantation window coincides with pervasive accessibility changes in decidualizing cells (68).

The gating function is most apparent in the hormonal response pathway. MLL1 catalyzes H3K4me3 at PGR regulatory regions, facilitating ERα-dependent transcriptional activation of PGR; its loss attenuates progesterone-driven decidualization despite intact hormone supply (6, 65). CFP1, which directs H3K4me3 placement genome-wide in uterine tissue, governs the broader epigenetic terrain upon which progesterone signaling depends (65). At the DNA level, HOXA10 promoter methylation silences a key receptivity gene; this methylation is reversible by hCG-mediated DNMT1 downregulation (30), closing the loop between hormonal input and epigenetic state. H3K27me3, deposited by EZH2, exerts the opposite gating effect, where enrichment of this repressive mark at decidualization loci blocks their activation even when progesterone signaling is intact (69, 70). The balance between activating (H3K4me3, H3K27ac) and repressive (H3K27me3, DNA methylation) marks at hormone-responsive loci therefore determines the effective threshold for progesterone action in any given cell.

Epigenetic gating extends equally to immune-relevant gene programs. miR-30b and miR-30d, upregulated in receptive-phase endometrium, target adhesion and immune signaling transcripts to fine-tune the WOI molecular environment (71, 72). SOCS1, the negative regulator of JAK-STAT inflammatory signaling, is itself under miRNA control during the WOI (73, 74). This arrangement lets post-transcriptional epigenetic regulation calibrate the intensity of immune signaling. circSTK40 modulates HSP90/AKT/FOXO1 and has been implicated in recurrent implantation failure, while miR-182-5p suppresses NDRG1 (75), interfering with the NF-κB/ZEB1/E-cadherin axis and disrupting epithelial-mesenchymal balance required for implantation. Aberrant miRNA and lncRNA profiles in endometriosis further impair hormonal and immune gene expression (76–79). Through these effects, non-coding RNAs set whether immune and adhesion programs reach their activation threshold before implantation.

Decidualization itself depends on epigenetic permission. TET3-mediated demethylation of Col1A1 and related targets facilitates stromal cell commitment to the decidual phenotype (80, 81). Loss of miR-29a impairs this process by disrupting TET3 function. N^6^-methyladenosine RNA modification provides an additional layer of post-transcriptional control over decidualization-associated transcripts (7, 70). The convergence of DNA methylation, histone marks, and ncRNA regulation on decidualization genes means that this developmental transition can proceed only when all of these controls permit it at once. A block in any one of them can prevent decidualization while other endometrial functions remain apparently intact.

So far this layer has been described as if chromatin states were fixed in advance, setting each response before any signal arrives. Evidence from Section 2 shows the relationship is not one-directional. Hormonal signals can themselves rewrite epigenetic marks. hCG suppresses DNMT1, and progesterone-dependent pathways recruit chromatin modifiers. The epigenetic layer therefore functions as more than a static precondition; the signals it gates also regulate it dynamically. This bidirectionality, in which hormones write epigenetic marks that then determine future hormonal responsiveness, creates the potential for both virtuous cycles, in which chromatin opens progressively during normal WOI establishment, and pathological lock-in, in which aberrant methylation prevents recovery of hormonal sensitivity. Whether immune signals similarly rewrite the endometrial epigenome, and whether such rewriting feeds back onto immune function itself, is the subject of the integrated coupling analysis that follows.

## 5 LIF as the converging node of the three-tier regulatory network and the coupling-decoupling model

The preceding sections have examined hormonal signaling, immune cell coordination, and epigenetic regulation as separate analytical threads. Evidence from each domain, however, repeatedly points beyond its own boundaries. For example, hCG modifies HOXA10 promoter methylation through DNMT1 downregulation (30), GH engages the LIF/JAK1/STAT3 axis shared with immune effectors, and CFP1-dependent H3K4me3 gates progesterone receptor transcription (65). These observations indicate that the three regulatory dimensions form a coupled architecture rather than parallel systems, one in which hormonal signals supply directional instructions, epigenetic states set the cellular response threshold, and immune effectors execute microenvironment remodeling while feeding back onto both upstream layers. This section synthesizes the available evidence into such an operating model and examines what occurs when specific coupling links are severed.

The coupling begins with hormonal signals leaving durable marks on the epigenome. As established in Section 4, hCG-driven DNMT1 downregulation sustains HOXA10 accessibility (30), while MLL1-catalyzed H3K4me3 keeps the PGR regulatory regions accessible (6) and CFP1 governs the broader H3K4me3 landscape on which the progesterone-PR axis depends (65). The integrative point is that the epigenetic layer sets a threshold for hormonal action rather than passively executing it. When aberrant methylation or histone-mark depletion raises this threshold, clinically normal hormone levels fail to produce adequate decidualization, a state of epigenetically driven progesterone resistance.

**Figure 4:**
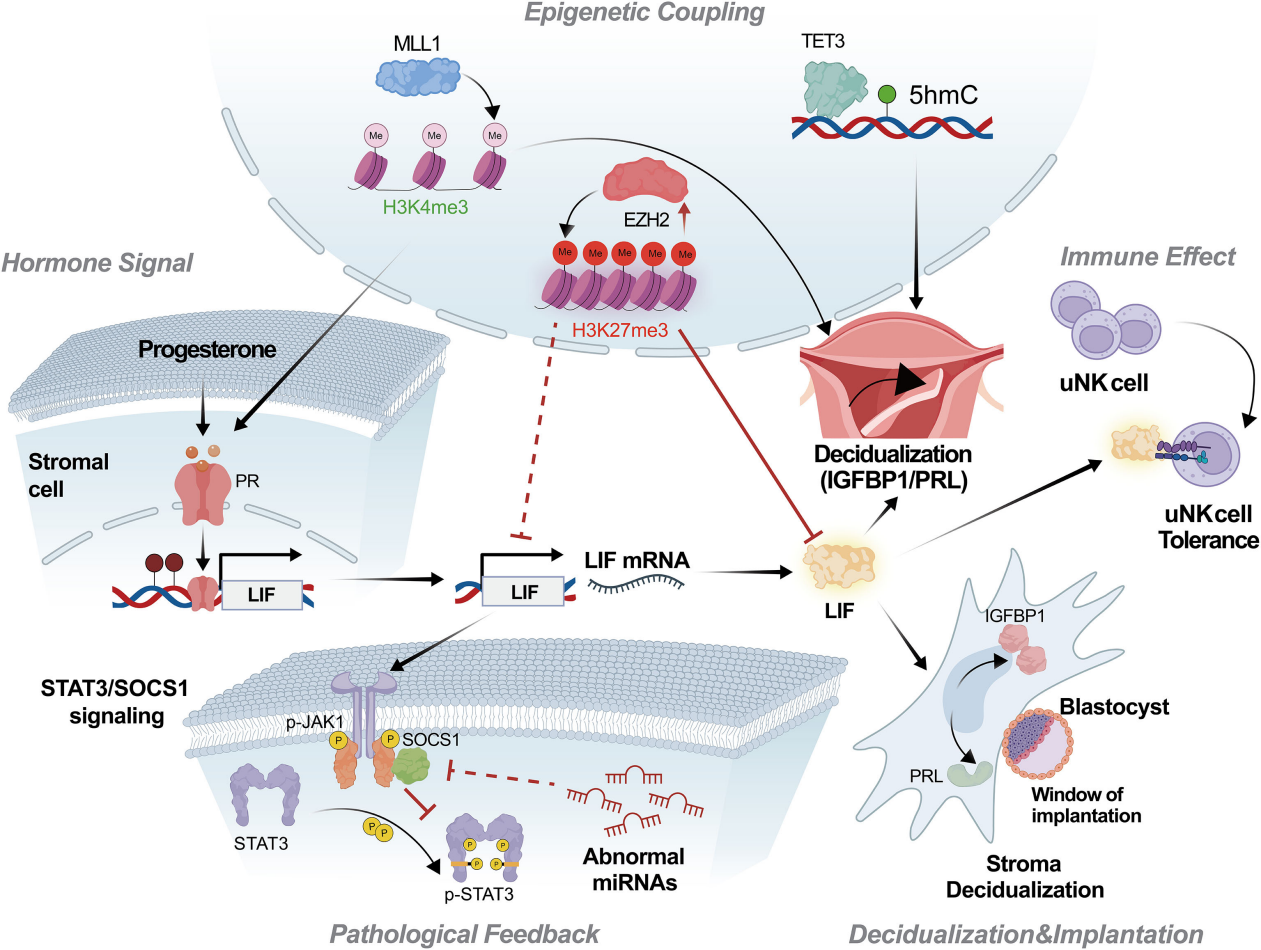
LIF as the convergence node of hormonal, immune, and epigenetic signaling in receptivity regulation. Progesterone-PR signaling in stromal cells induces LIF expression, by which decidualization is promoted and uNK cell-mediated immune tolerance is modulated. Epigenetic coupling through MLL1, EZH2, and TET3 regulates transcriptional accessibility of hormone-responsive and decidualization-related loci. JAK1/STAT3 signaling activated by LIF is constrained by SOCS1 negative feedback, with this regulatory loop potentially perturbed by dysregulated miRNAs.

LIF was discussed in preceding sections from two vantage points, first as a downstream target of progesterone-PR signaling (Section 2) and as a cytokine that shapes uNK and macrophage behavior at the decidual interface (Section 3). Integrating these observations positions LIF as the principal molecular node through which hormonal instructions are converted into immune microenvironment directives (**Figure 4**). Progesterone and hCG induce LIF expression predominantly in endometrial epithelial and stromal cells, with uNK cells contributing a second hormone-responsive source as noted in Section 3 (82–84). Once secreted, LIF activates JAK1/STAT3 in target immune populations, promoting the tolerogenic phenotype of uNK cells and supporting trophoblast invasion (63, 85, 86). GH reinforces this relay by upregulating LIF while engaging SOCS1-mediated negative feedback that prevents unchecked inflammatory amplification. In RIF patients whose PR expression remains within reference ranges, LIF is nonetheless significantly reduced (74, 87, 88), indicating that the hormonal signal can exit the nucleus intact yet fail at the step of immune translation. This selective uncoupling shows that the hormone-to-immune link can be disrupted independently of classical hormone deficiency.

The relationship between epigenetic regulation and immune function runs in both directions, forming a self-reinforcing loop. In the forward direction, EZH2-deposited H3K27me3 silences genes required for immune tolerance at the decidual interface; TET3-mediated demethylation of targets such as Col1A1 facilitates stromal cell differentiation into a decidual phenotype capable of supporting local immune homeostasis (80, 81). miRNA-mediated post-transcriptional control provides additional fine-tuning, since SOCS1, the negative regulator of JAK-STAT inflammatory signaling, is itself subject to miRNA modulation during the WOI, which ties epigenetic control directly to immune signal calibration (74, 79, 89–94).

In the reverse direction, pro-inflammatory cytokines reshape the endometrial epigenome. IL-1β and TNF-α alter ncRNA profiles in endometrial stromal cells, and macrophage-derived exosomal miR-22-3p suppresses SIRT1 to activate NF-κB in recipient stromal cells (90). IL-1β additionally depletes PR-A and PR-B protein through an ERK1/2-dependent mechanism, effectively erasing hormonal responsiveness at the protein level (91). TNF-α shifts the steroid receptor landscape by suppressing PR while upregulating glucocorticoid receptors (92), and chronic B-cell-driven inflammation in endometritis paradoxically elevates PR transcript levels while abolishing functional decidualization (93). The bidirectional nature of this loop carries a critical implication. Once inflammatory signals have rewritten the local epigenetic state, removal of the initial immune trigger may not restore normal responsiveness, because the epigenetic imprint of inflammation persists (94). Conversely, epigenetic lesions that silence immune-tolerance genes can initiate chronic low-grade inflammation that further entrenches the aberrant chromatin state.

If the three-dimensional coupling model is correct, distinct pathological states should map to specific decoupling sites rather than to generalized system failure. Available evidence supports this prediction (4). In endometriosis, EZH2 overexpression drives aberrant H3K27me3 accumulation that silences decidualization-related and immune-tolerance genes (16, 77, 95, 96); the primary lesion is an epigenetic-to-immune decoupling in which chromatin compaction prevents normal immune reprogramming. In women of advanced reproductive age, genome-wide reduction of H3K27ac accompanies attenuated PR signaling and diminished expression of IGFBP1 and FOXO1 (97); here the dominant failure is an epigenetic-to-hormonal decoupling whereby depleted activating histone marks raise the threshold for progesterone action. In a subset of RIF patients, LIF suppression coexists with preserved PR expression (49, 87, 88), and the defect localizes to the hormone-to-immune junction where endocrine output fails to be translated into cytokine signaling. Each pattern produces implantation failure, yet each originates from a different node in the coupled network. A uniform diagnostic or therapeutic strategy applied across these mechanistically distinct presentations will inevitably fail for the majority of patients whose decoupling site falls outside the single dimension being addressed.

The heterogeneity of decoupling sites carries a direct implication for clinical assessment, in that no single-dimension tool can identify which coupling link has failed in a given patient. Evaluation systems must therefore be organized by the regulatory dimension they interrogate, and their clinical value reappraised through the lens of dimensional coverage rather than sensitivity to a universal receptivity marker.

## 6 Assessment methods for endometrial receptivity and translational applications under multidimensional regulation

The coupled network model developed in Section 5 predicts that different patients experience receptivity failure at different decoupling sites. An assessment system aligned with this model must therefore identify which regulatory dimension is primarily compromised in a given individual (4). Current clinical tools, however, were developed before the multi-dimensional framework existed and were validated against pregnancy outcome rather than against specific mechanistic targets. The following discussion reorganizes available assessment modalities by the regulatory dimension they interrogate, exposing both their mechanistic rationale and their dimensional blind spots.

Tools that primarily reflect hormonal-axis status form the oldest and most widely validated tier of receptivity assessment. Endometrial thickness and Doppler blood flow indices measure tissue-level consequences of estrogen-driven proliferation and progesterone-driven vascular remodeling; they remain the clinical foundation due to convenience and repeatability, though inconsistent predictive value for pregnancy outcomes has limited their standalone utility (8, 98–100). Ultrasound elastography quantifies tissue mechanical properties that reflect stromal decidualization and extracellular matrix remodeling downstream of progesterone signaling (13, 101). At the molecular level, transcriptomic platforms such as the endometrial receptivity analysis (ERA), endometrial receptivity map (ERMap), and beREADY capture gene expression signatures that largely represent the hormonal-transcriptomic state of the endometrium (102, 103). The most rigorous clinical test of this approach, a randomized controlled trial enrolling 767 patients, found no improvement in live birth rates when ERA-guided personalized transfer timing was compared with standard timing (104). Subsequent meta-analyses confirmed this null result across broader populations (105–107). Single molecular markers assessed by immunohistochemistry, including integrin αvβ3, LIF, and HOXA10, also target hormone-responsive gene products; despite mechanistic relevance, high inter-study variability has prevented clinical standardization (108, 109). The shared limitation across this tier is dimensional. All these tools interrogate downstream readouts of hormonal signaling, leaving immune and epigenetic dimensions unexamined.

A second group of assessment modalities targets the immune-inflammatory dimension. Chronic endometritis, identified by CD138-positive plasma cell infiltration, is a directly modifiable immune pathology affecting receptivity; diagnosis combines hysteroscopic evaluation with immunohistochemical confirmation (94, 110, 111). Endometrial microbiome analysis through next-generation sequencing addresses the microbial-immune interface, where dominance of Lactobacillus has been associated with favorable reproductive outcomes, while dysbiotic profiles correlate with inflammatory activation (112–114). These tools capture immune status more directly than transcriptomic platforms, yet they assess isolated aspects of the immune dimension (chronic infection, microbial composition) rather than the integrated immune-tolerance program described in Section 3.

Emerging liquid biopsy approaches hold potential for multi-dimensional sampling. Uterine fluid proteomic profiling has identified inflammatory markers associated with receptivity status (115, 116). Extracellular vesicles isolated from uterine fluid carry transcriptomic cargo, including small non-coding RNAs, that may reflect epigenetic regulatory states of the source tissue, representing the closest available approximation to non-invasive epigenetic-dimension assessment (10–12, 117, 118). Seminal fluid-derived high-density extracellular vesicles have been shown to enhance endometrium-trophoblast adhesion through upregulation of LIF and STAT3 activation (85), a finding that connects this research to the hormone-immune convergence node identified in Section 5. Liquid biopsy approaches remain investigational; standardization of collection protocols and establishment of reference ranges require prospective validation (115). Their conceptual advantage, however, is access to molecular information from multiple regulatory layers simultaneously without requiring tissue biopsy.

Current assessment methods are summarized in **Table 1**, stratified by methodological category and evidence summary. When viewed through the dimensional lens, the table reveals a structural imbalance. Validated tools cluster heavily in the hormonal dimension, immune assessment remains limited to infection markers, and epigenetic-level evaluation exists only in research settings. No single tool or platform currently provides coverage across all three dimensions. Integration of artificial intelligence (AI) into imaging assessment offers improved standardization (automatic endometrial segmentation, objective thickness quantification) but does not address this dimensional gap, as AI-enhanced imaging still interrogates hormone-dependent morphological features (119, 120).

**Table 1:** Assessment methods for endometrial receptivity.

Category	Method	Sample	Key indicators	Advantages	Limitations	Evidence summary
Imaging	TVS 2D basic parameters	Non-invasive	Endometrial thickness, echogenicity	Non-invasive, convenient, repeatable	Inconsistent predictive value	Meta-analyses; ESHRE recommended
	Doppler ultrasound	Non-invasive	PI, RI, blood flow grading	Non-invasive, reflects perfusion	Operator-dependent, non-standardized thresholds	Meta-analysis negative; no guideline
	3D Power Doppler	Non-invasive	Endometrial volume, VI/FI/VFI	Quantitative vascularization assessment	Equipment-dependent, limited generalizability	Meta-analysis positive; no guideline
	Ultrasound elastography	Non-invasive	Strain ratio, stiffness indices	Reflects tissue mechanical properties	Single-center data only, requires validation	Prospective cohorts; investigational
Structural	Hysteroscopy	Endoscopy	Polyps, adhesions, inflammation	Direct structural visualization; guideline-recommended for RIF	Invasive, indirect receptivity measure	RCT negative; ESHRE recommended for pathology
Histological	Noyes criteria dating	Biopsy	Glandular/stromal staging	Classic, parallel validation	Subjective, limited reproducibility, largely abandoned	Prospective study negative; ASRM against
	Pinopode detection	Biopsy/EM	Pinopode timing/density	Morphologically intuitive	Broad window, controversial consistency	Single RCT; no guideline, investigational
Molecular markers	Single IHC markers	Biopsy	Integrin αvβ3, LIF, HOXA10	Mechanistically relevant	High variability, non-standardized	Case-control only; no guideline
Immune phenotype	BCL6 expression	Biopsy/IHC	HSCORE	Links inflammation to outcomes	Indirect relationship, varies by preparation method	Prospective negative; no guideline
Infection	CE assessment	Biopsy/Pathology	CD138+ plasma cells	Identifies modifiable etiology	Non-standardized criteria	Meta-analyses positive; ESHRE conditionally recommended
Transcriptomic	ERA	Biopsy/Transcriptomics	Receptive/Non-receptive, WOI shift	Individualized timing	Invasive, costly, disputed benefit	RCT no benefit; NICE against routine use
	ERMap	Biopsy/Gene panel	Receptivity prediction	Clinically feasible	Limited validation	Prospective validation; no guideline
	beREADY (TAC-seq)	Biopsy/Targeted seq	Targeted gene panel	Sensitive, novel approach	Prospective data accumulating	Prospective validation; no guideline
Microbiome	EMMA/ALICE	Endometrial/NGS	Microbial composition	Guides intervention	Contamination risk, limited standardization	Prospective supportive; ESHRE against
Liquid biopsy	UF proteomics	Uterine fluid	Protein/inflammatory profiles	Approaching non-invasive goal	Methodological variations	Discovery-validation; no guideline
	UF-EVs multi-omics	Uterine fluid EVs	EV-RNA/proteins	Potential biopsy alternative	Research stage, standardization needed	Prospective pilot (AUC 0.88); no guideline

AUC, area under the curve; CE, chronic endometritis; EM, electron microscopy; ERA, Endometrial Receptivity Analysis; ERMap, endometrial receptivity map; EVs, extracellular vesicles; FI, Flow Index; IHC, immunohistochemistry; NGS, next-generation sequencing; PI, Pulsatility Index; RI, Resistance Index; seq, sequencing; TVS, transvaginal sonography; UF, uterine fluid; VI, Vascularization Index; VFI, Vascularization Flow Index; WOI, window of implantation.

Evidence Summary column reports the highest level of published evidence (RCT, meta-analysis, prospective cohort, case-control, or discovery-validation study) and guideline recommendation status (ESHRE, ASRM, or NICE) for each method.

Doppler ultrasound assessment includes uterine artery, endometrial, and subendometrial blood flow evaluation.

Single IHC markers include integrin αvβ3, LIF, and HOXA10, among others.

CE assessment typically combines CD138 immunostaining with hysteroscopic evaluation.

The dimensional analysis above reveals a structural mismatch between available assessment tools and the multi-dimensional nature of receptivity failure. Closing it requires a stratification logic that matches diagnostic modality to the patient-specific decoupling site, a challenge addressed in the Discussion.

## 7 Discussion

Translation of the multi-dimensional coupling model into clinical action requires confronting a central paradox, namely that the most rigorously tested molecular receptivity tool has not improved pregnancy outcomes. The ERA trial enrolled 767 patients and found no live birth benefit from personalized transfer timing guided by endometrial transcriptomics (104). Two subsequent meta-analyses confirmed this null result (105, 106), and a critical reanalysis argued that ERA-guided transfer may actually reduce success rates (121). The mechanistic premise of ERA is sound, since gene expression patterns do shift across the WOI, and displaced windows have been documented in RIF subsets (122). The disconnect between mechanistic validity and clinical futility becomes explicable, however, once ERA is recognized as a single-dimension tool applied to a multi-dimensional problem. The transcriptome is, admittedly, the integrated output of all three regulatory tiers, and expression shifts do carry the imprint of upstream epigenetic and immune events. This very convergence is the source of the limitation. ERA classifies a sample against a fixed transcriptomic signature calibrated to the timing of the window rather than to its regulatory cause, so it reports whether the endometrium is pre-receptive, receptive, or post-receptive without resolving which upstream layer produced that state. A displaced window arising from epigenetic progesterone resistance and one arising from delayed hormonal exposure can yield overlapping signatures yet demand opposite interventions. Because its readout is a phase call rather than a layer-resolved one, ERA cannot localize immune decoupling (abnormal LIF despite normal PR), epigenetic gating failures (H3K27me3 silencing of decidualization genes), or the inflammatory rewriting of chromatin states described in Section 5. In an unselected RIF population where decoupling sites are heterogeneous, ERA will correctly identify the minority (107) whose failure is purely hormonal-transcriptomic while missing the remainder. Dilution of a true positive signal across a mixed population produces the observed null result.

This interpretation generates a testable prediction. ERA should perform differently in patient subgroups pre-stratified by decoupling phenotype. Recent evidence supports the premise of such stratification. Transcriptomic and metabolomic clustering has identified molecularly distinct RIF subtypes with divergent pathway activity and immune infiltration patterns (4, 123). Integrated multi-omics analyses combining metabolomics, proteomics, single-cell RNA-seq, and spatial metabolomics have revealed that RIF involves epithelial metabolic and adhesive dysregulation invisible to transcriptomics alone (124, 125). These findings confirm that RIF behaves as a heterogeneous syndrome whose constituent subtypes differ in their primary decoupling site, well beyond any single disease entity.

Building on this evidence, we propose a conceptual stratification framework organized by decoupling phenotype. Whereas Section 5 illustrated three representative decoupling sites as mechanistic exemplars, clinical stratification instead requires operational categories, comprising three single-dimension-dominant phenotypes and a mixed phenotype for concurrent failures. A hormone-dominant phenotype would be characterized by displaced WOI or progesterone resistance detectable by transcriptomic profiling; these patients are the appropriate candidates for ERA-guided transfer timing or hormonal supplementation optimization. An immune-dominant phenotype would present with chronic endometritis, elevated inflammatory cytokines, or abnormal uNK subpopulation ratios; detection requires CD138 immunohistochemistry, uterine fluid cytokine panels, or microbiome analysis (114), and intervention targets the inflammatory state rather than transfer timing. An epigenetic-dominant phenotype would manifest as progesterone resistance with normal PR expression but aberrant histone marks or miRNA profiles; uterine fluid extracellular vesicle cargo may serve as a non-invasive readout (10–12) for this category. A mixed or network-collapse phenotype, in which multiple coupling links fail simultaneously, would require combinatorial assessment and multi-target intervention. This framework remains hypothetical and requires prospective validation, but it provides a mechanistically grounded basis for trial design that current empirical stratification approaches lack.

Matching intervention to decoupling phenotype reframes existing therapeutic evidence. Chronic endometritis treatment with antibiotics addresses an immune-layer pathology, yet persistence of early pregnancy loss risk after microbiological cure (94) may reflect residual epigenetic damage inflicted by prior inflammation, consistent with the inflammatory memory mechanism described in Section 5. Intrauterine platelet-rich plasma (PRP), whose growth factor content addresses multiple pathways simultaneously (126, 127), may derive whatever benefit it offers from its multi-dimensional rather than single-target nature. miRNA-124-3p, which negatively regulates both LIF and MUC1 (86), operates at the epigenetic-immune interface and could represent a candidate therapeutic target for the epigenetic-dominant phenotype. These reinterpretations do not invalidate existing interventions but suggest that their efficacy should be re-evaluated within phenotype-stratified populations rather than in unselected RIF cohorts.

Validation of phenotype-based stratification requires experimental systems capable of modeling multi-dimensional interactions. *In vitro* embryo-endometrium interface models and three-dimensional receptive endometrium organoids now recapitulate key molecular interactions between trophoblast and decidualized stroma (128, 129). Single-cell transcriptomic atlases of luteal-phase endometrium have mapped cell-type-specific signaling dynamics with temporal resolution (44), so that decoupling events become identifiable at the single-cell level. Animal models, particularly conditional knockout systems targeting specific coupling nodes (MLL1, CFP1, EZH2), provide causal evidence that cannot be obtained in human observational studies (6, 65, 69). The convergence of organoid technology, spatial transcriptomics, and uterine fluid liquid biopsy creates a realistic path toward clinical implementation of phenotype-stratified receptivity assessment within the coming decade.

Several limitations warrant acknowledgment. As a narrative synthesis, this work reflects interpretation of selected literature rather than systematic evaluation of all available evidence. The proposed coupling model and stratification framework are conceptual constructs derived from indirect evidence across multiple study systems; direct demonstration of bidirectional causality between dimensions remains limited to a small number of molecular nodes. Much of the cited mechanistic evidence derives from *in vitro* or animal models whose translation to human endometrial physiology requires caution. The proposed patient phenotypes have not been validated in prospective clinical cohorts, and the boundaries between subtypes are likely blurred rather than discrete. Finally, publication bias toward positive findings in the receptivity field may inflate the apparent strength of certain mechanistic links.

## 8 Conclusion

The framework presented here recasts implantation failure as a problem of inter-dimensional coupling rather than single-pathway deficiency. This review has argued that these three dimensions form a single operating system rather than three parallel pathways, with LIF, histone modifiers, and inflammatory cytokines serving as molecular coupling nodes. When specific coupling links are severed, the resulting implantation failure maps to a specific decoupling site, whether epigenetic-to-immune (endometriosis), epigenetic-to-hormonal (reproductive aging), or hormone-to-immune (a subset of RIF). The clinical consequence is that single-dimension assessment tools, including transcriptomic platforms validated in unselected populations, cannot identify the affected dimension in a given patient. Progress toward precision management of implantation failure will require phenotype-stratified trial designs that match diagnostic and therapeutic interventions to the patient-specific decoupling site. Uterine fluid liquid biopsy, spatial transcriptomics, and multi-omics integration offer technically feasible paths toward this goal, though prospective validation remains the critical next step.
